# PML‐RARα interferes with erythropoiesis by repressing *LMO2* in acute promyelocytic leukaemia

**DOI:** 10.1111/jcmm.13917

**Published:** 2018-10-15

**Authors:** Xianwen Yang, Yun Tan, Ping Wang, Hui Zhang, Ming Zhao, Xujie Zhao, Kankan Wang

**Affiliations:** ^1^ State Key Laboratory of Medical Genomics and Shanghai Institute of Hematology Ruijin Hospital Shanghai Jiao Tong University School of Medicine Shanghai China; ^2^ Sino‐French Research Center for Life Sciences and Genomics Ruijin Hospital Shanghai Jiao Tong University School of Medicine Shanghai China

**Keywords:** acute promyelocytic leukaemia, erythropoiesis, LMO2, PML‐RARα

## Abstract

The *PML‐RAR*α fusion gene, generated by the t(15;17) chromosome translocation, is regarded as the initiating factor of acute promyelocytic leukaemia (APL). In addition to the well‐known effects on blocking myeloid differentiation at the promyelocytic stage, promyelocytic leukaemia‐retinoic acid receptor α (PML‐RARα) has also been reported to interfere with multiple differentiation processes, including erythroid differentiation. However, the detailed molecular mechanism by which PML‐RARα impairs erythropoiesis has not yet been fully addressed. By chromatin immunoprecipitation‐PCR assay, we found that PML‐RARα bound to the distal promoter region of *LMO2* (LIM‐only protein 2), a critical erythroid‐specific transcription factor. Luciferase reporter assays and qRT‐PCR results demonstrated that PML‐RARα down‐regulated the expression of the *LMO2* distal transcript through transrepressing its promoter activity. Analysis of gene expression profiling data from large cohorts of acute myeloid leukaemia (AML) patients confirmed that *LMO2* expressed at a markedly lower level in APL patients in comparison to non‐APL AML patients. Further flow cytometry analysis demonstrated that PML‐RARα inhibited erythropoietin‐induced erythroid differentiation by down‐regulating *LMO2* expression. Our findings reveal a previously unidentified mechanism, by which PML‐RARα interferes with erythropoiesis through directly targeting and transrepressing *LMO2* expression in the development of APL.

## 1 INTRODUCTION

Acute promyelocytic leukaemia (APL), a subtype of acute myeloid leukaemia (AML), is characterized by the t(15;17)(q22;q21) chromosomal translocation. The resultant fusion protein promyelocytic leukaemia‐retinoic acid receptor α (PML‐RARα) is well‐known to be responsible for a differentiation block at the promyelocytic stage,^1^, ^2^ resulting in the aberrant accumulation of immature promyelocytes in bone marrow and peripheral blood. Leukaemia initiating cells (LICs) in APL have been reported from the different models. Some studies regard that APL LICs are myeloid committed cells, based on the transgenic mouse models in which PML‐RARα expression is under control of more differentiated myeloid specific promoters.^3^, ^4^, ^5^ Interestingly, other studies have also indicated that PML‐RARα is expressed at the early stage of hematopoietic hierarchy such as multipotent progenitors rather than committed myeloid progenitors and promyelocytes only,^6^, ^7^, ^8^ indicating that the influence of PML‐RARα may not be limited to myeloid cells but other lineages of blood cells as well. Furthermore, PML is consistent with the previous finding in early hematopoiesis and erythropoiesis,^9^ suggesting that the disrupted expression pattern of PML by PML‐RARα may affect normal erythropoiesis. Indeed, it has been reported that PML‐RARα can interfere with hemin‐induced erythroid differentiation in K562 cells,^10^ further supporting the idea that PML‐RARα may impair erythropoiesis. However, the molecular mechanism by which PML‐RARα influences erythroid differentiation is not yet clear.

LMO2 (LIM‐only protein 2, also known as RBNT2), is an important regulator of hematopoietic stem cell development and erythropoiesis, as mice deficient in *Lmo2* show a complete lack of blood cells and defects in the formation of foetal erythrocytes.^11^ LMO2 has been demonstrated to function as a bridge molecule and assist in the assembly of multimeric transcription factor complexes. LMO2 is capable of inducing erythroid differentiation through the interaction with transcription factors, including SCL, E2A, LDB1 and GATA‐1.^12^, ^13^ Such a transcriptional complex regulates the expression of erythroid‐specific genes, such as the α*‐globin* genes,^14^ *EKLF* 15 and *glycophorin A* (*GPA*).^16^ Knockdown of LMO2 results in the disassembly of this transcriptional complex and thereby attenuates the chromatin occupancy of GATA‐1 and LDB1,^17^ ultimately leading to the dysregulated expression of erythroid‐specific genes. Moreover, forced expression of LMO2 is able to rescue the defective erythroid differentiation caused by c‐myb silencing in CD34 positive cells.^18^ The above findings indicate the important role of *LMO2* in erythropoiesis.

In the present work, we found that PML‐RARα but not wild‐type RARα bound to the distal promoter of *LMO2* and thereby down‐regulated the expression of *LMO2* through decreasing the promoter activity. We showed that *LMO2* expression was significantly lower in APL patients than that in non‐APL AML patients. Functionally, *LMO2* expression was up‐regulated in umbilical cord blood (UCB)‐derived CD34 positive cells upon erythropoietin (EPO)‐induction of erythropoiesis. Forced expression of PML‐RARα into the CD34 positive cells arrested EPO‐induced erythropoiesis by repressing *LMO2* expression. Taken together, our results demonstrated that PML‐RARα interfered with erythroid differentiation through directly targeting the *LMO2* distal transcript and repressing *LMO2* expression in the pathogenesis of APL.

## 2 MATERIALS AND METHODS

### 2.1 Cell lines culture

U937‐PR9 was a gift from Dr. PG Pelicci (Milan, Italy). NB4 was a gift from Dr. M Lanotte (Hospital St Louis, Paris, France). 293T was obtained from the Cell Bank at the Chinese Academy of Sciences (Shanghai, China). U937‐PR9 and NB4 cells were maintained in RPMI 1640 (Gibco, Carlsbad, CA, USA) each containing 10% foetal bovine serum (FBS) (Gibco). 293T cells were cultured in DMEM (Gibco) containing 10% FBS. Cells were cultured in an incubator at 37°C with 5% CO_2_. ZnSO_4_ (Sigma, St. Louis, MO, USA) was used to induce the expression of PML‐RARα in U937‐PR9 cells at the final concentration of 100 μM. Both all‐*trans* retinoic acid (ATRA) (Sigma) and arsenic trioxide (ATO) (Sigma) were dissolved in absolute ethanol and MQ water respectively. ATRA and ATO are used at the final concentration of 1 μM.

### 2.2 Human UCB specimens

The study was approved by the Ethics Committee of Ruijin Hospital affiliated to Shanghai Jiaotong University School of Medicine and was adherent to the regulation of the declaration of Helsinski. The approval number is ChiCTR‐OPC‐15006492. Fresh human UCB specimens were obtained from volunteer donors attending obstetrics department at Ruijin Hospital. Informed consent was obtained according to institutional guidelines.

### 2.3 Isolation of UCB‐derived CD34 positive cell and stimulation of erythroid differentiation in vitro

CD34 positive cells were isolated by Ficoll (Axis‐Shield, Oslo, Norway) density centrifugation for mononuclear cells and subsequent magnetic cell sorting for cells stained with anti‐CD34 antibody (Becton Dickinson, Franklin Lakes, NJ, USA). Freshly isolated CD34 positive cells were cultured in the Serum‐Free expansion Medium (StemCell Techologies, Vancouver, BC, Canada) containing 40 ng/mL of granulocyte‐macrophage colony‐stimulating factor (Baote Biology Co., Ltd, China), 20 ng/mL of interleukin‐3 (Sigma) and 100 ng/mL of stem cell factor (Sigma). EPO (Sansheng Pharmaceutical Co., Ltd, Shenyang, China) was added to stimulate cell differentiate along with the erythroid lineage at the final concentration of 5 IU/mL. The cells were collected at a series of time‐points after treatment. These cells were immunostained with CD235a antibody (Becton Dickinson) and subsequently analysed by flow cytometry (Becton Dickinson).

### 2.4 RNA extraction and RT‐PCR

Total RNA of leukaemic cell lines and UCB‐derived CD34 positive cells with/without manipulation were extracted using the TRIzol reagent (Invitrogen, Carlsbad, CA, USA) and RNeasy Micro Kit (Qiagen, Santa Clarita, CA, USA) respectively. cDNA was converted using the SuperScript II Reverse Transcriptase (Invitrogen) with random hexamer primers according to the manufacturer's protocol. RT‐PCR was performed to measure the mRNA levels of *PML‐RAR*α and *LMO2*. *Glyceraldehyde 3‐phosphate dehydrogenase* (*GAPDH*) was used as an internal control. The information of primer sequences is as follows, LMO2‐F: 5′‐CAAAGCAGGCAATTAGCCC‐3′; LMO2‐R: 5′‐CCTCTCCACTAGCTACTGC‐3′; PML‐RARα‐F: 5′‐AAGTGAGGTCTTCCTGCCCAA‐3′; PML‐RARα‐R: 5′‐GGCTGGGCACTATCTCTTCAGA‐3′; GAPDH‐F: 5′‐GAAGGTGAAGGTCGGAGTC‐3′; GAPDH‐R: 5′‐GAAGATGGTGATGGGATTTC‐3′; each experiment was performed in triplicate.

### 2.5 Plasmid construction, transient transfection and luciferase assays

The *LMO2* distal promoter regions including both the full length (approximately 2.3 kb upstream of the *LMO2* transcription start site) and truncated form were cloned into PGL3‐basic vector (Promega, Madison, WI, USA) respectively. Plasmids were transfected into 293T cells using Lipofectamine 2000 (Invitrogen) according to the manufacturer's instructions. UCB‐derived CD34 positive cells were transfected using Amaxa Human CD34+ cell Nucleofector Kit (Amaxa, Cologne, Germany). Luciferase assays were performed with Dual‐Luciferase Reporter Assay (Promega) 48 hours after transfection. Briefly, the transfected 293T cells were lysed with passive lysis buffer (Promega) and 10 μL of cell lysate was aspirated for measurement. Luciferase activities were normalized by cotransfecting a plasmid expressing Renilla luciferase. The Primers for luciferase constructs are as follows, *LMO2*‐full length‐F: 5′‐ccgctcgagCTGACACAGATAACCCCTCAAG‐3′; *LMO2*‐full length‐R: 5′‐cccaagcttGATGTGCTCTGCGTGGAATC‐3′; *LMO2*‐truncated‐F: 5′‐ccgctcgagCCTCCTTGCAAAGTGAGAAGG‐3′; *LMO2*‐truncated R is the same with *LMO2*‐full length R; *LMO2*‐RAREh (1st mutation) F: 5′‐GCTGTGGGTAAGCAGGTCCAATGctttagCAATTTTACATTGAGA‐3′; *LMO2*‐RAREh (1st mutation) R: 5′‐TCTCAATGTAAAATTGctaaagCATTGGACCTGCTTACCCACAGC‐3′; *LMO2*‐RAREh (2nd mutation) F: 5′‐CAGAGAGTCTTACCActttagAGGGATTTAGAGAGGATCGAAGAG‐3′; *LMO2*‐RAREh (2nd mutation) R: 5′‐CTCTTCGATCCTCTAAATCCCTctaaagTGGTAAGACTCTCTG‐3′.

### 2.6 Chromatin immunoprecipitation analysis

Chromatin immunoprecipitation (ChIP) assays were performed according to the Affymetrix protocol as described,^19^ with the following antibodies: anti‐RARα (C‐20 X; Santa Cruz Biotechnology, Santa Cruz, CA, USA), anti‐PML (H238 X; Santa Cruz Biotechnology) and the rabbit immunoglobulin G (ab46540; Abcam, Cambridge, UK). PCR was performed to detect the enrichment. Each experiment was performed in triplicate and equivalent results were observed. Promoter primers used for ChIP‐PCR are as follows: LMO2‐DP‐F: 5′‐GCACTTATAACTGTTCAGACC‐3′; LMO2‐DP‐R: 5′‐CCAATGCTATGTAACACACAC‐3′; LMO2‐N‐F: 5′‐GGTGAGTGATGCTGCCTAAACC‐3′; LMO2‐N‐R: 5′‐ACTGAGATATCTGGGGAAGAGCA‐3′.

### 2.7 Gene expression analysis

Three transcriptome data sets of AML patients, including TCGA,^20^ GSE10358^21^ and GSE1159,^22^ were used to compare the expression of *LMO2* between APL and non‐APL patient samples. To perform interarray comparison, the CEL files were analysed by Affymetrix MAS 5.0 software (Affymetrix, Santa Clara, CA, USA). Two‐tailed *t*‐tests were used to validate the significance of the observed differences, which were considered statistically significant when *P* < 0.05.

### 2.8 Gene Ontology analysis

ChIP‐Seq data set GSM552237^23^ by using Lmo2 antibody in mouse hematopoietic progenitor cell line (HPC‐7) was retrieved to investigate the downstream target genes of *Lmo2*. To compare the expression of the *Lmo2* targets genes between human AML samples, conversion of the genomic co‐ordinates from mouse to human orthology was performed based on the Mouse Genomic Informatics database. Gene Ontology (GO) analysis was performed on differentially expressed *LMO2* target genes by using the ClueGO of the Cytoscape software including the following databases: Kyoto Encyclopedia of Genes and Genomes (KEGG), GO Molecular Function, GO Cellular Component and GO biological Process. The *P*‐values denote the significance of GO terms enrichment. The *P*‐value <0.05 is considered statistically significant.

## 3 RESULTS

### 3.1 PML‐RARα binds to the distal promoter of *LMO2*

**Figure 1:**
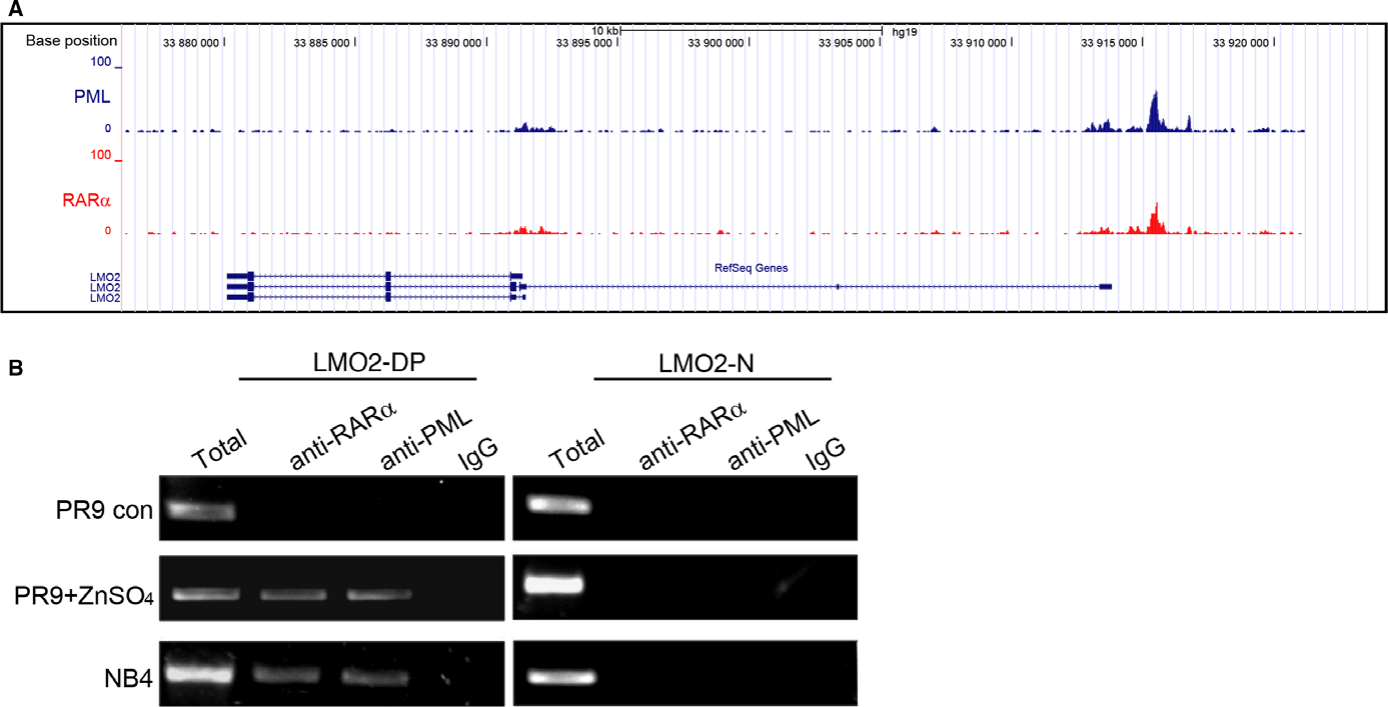
PML‐RARα binds to the distal promoter of *LMO2*. (A) Schematic diagram showing the binding of PML‐RARα to the distal promoter regions of *LMO2*. ChIP assays were performed in the PML‐RARα‐inducible PR9 cells using anti‐RARα and anti‐PML antibodies. The peaks represent the PML‐RARα‐enriched ChIP regions. (B) PML‐RARα bound to the distal promoter of *LMO2* in PML‐RARα‐inducible PR9 cells and APL patient‐derived NB4 cells. ChIP was performed with anti‐RARα, anti‐PML or normal immunoglobulin G (IgG) antibodies. ChIP‐PCR was performed with primers specific for the distal promoter region of *LMO2* (LMO2‐DP) or a non‐relevant region far from the *LMO2* locus (LMO2‐N). Total DNA or chromatin DNA immunoprecipitated with different antibodies was used for PCR amplification

To identify the potential genes that might be involved in the inhibition of erythroid differentiation in the pathogenesis of APL, we screened the PML‐RARα targets that we previously discovered from genome‐wide studies.^24^ Interestingly, we found that PML‐RARα was significantly enriched in the distal promoter region of *LMO2* (Figure 1A). Three alternative transcripts of *LMO2* have been identified so far, among which the distal promoter is regarded as an erythroid‐specific promoter due to the direct regulation by GATA‐1.^25^ To verify the PML‐RARα binding on the distal promoter of *LMO2*, we performed ChIP‐PCR assays in ZnSO_4_‐treated PR9 cells and APL patient‐derived NB4 cells using anti‐PML and anti‐RARα antibodies. As illustrated in Figure 1B, the positive bands were only amplified in the ChIPed region in ZnSO_4_‐treated PR9 cells and NB4 cells but not in untreated PR9 cells. These results indicate that PML‐RARα rather than wild‐type RARα binds to the distal promoter of *LMO2* in APL cells.

### 3.2 PML‐RARα down‐regulates the expression of *LMO2* through transcriptional repression of the *LMO2* distal transcript

**Figure 2:**
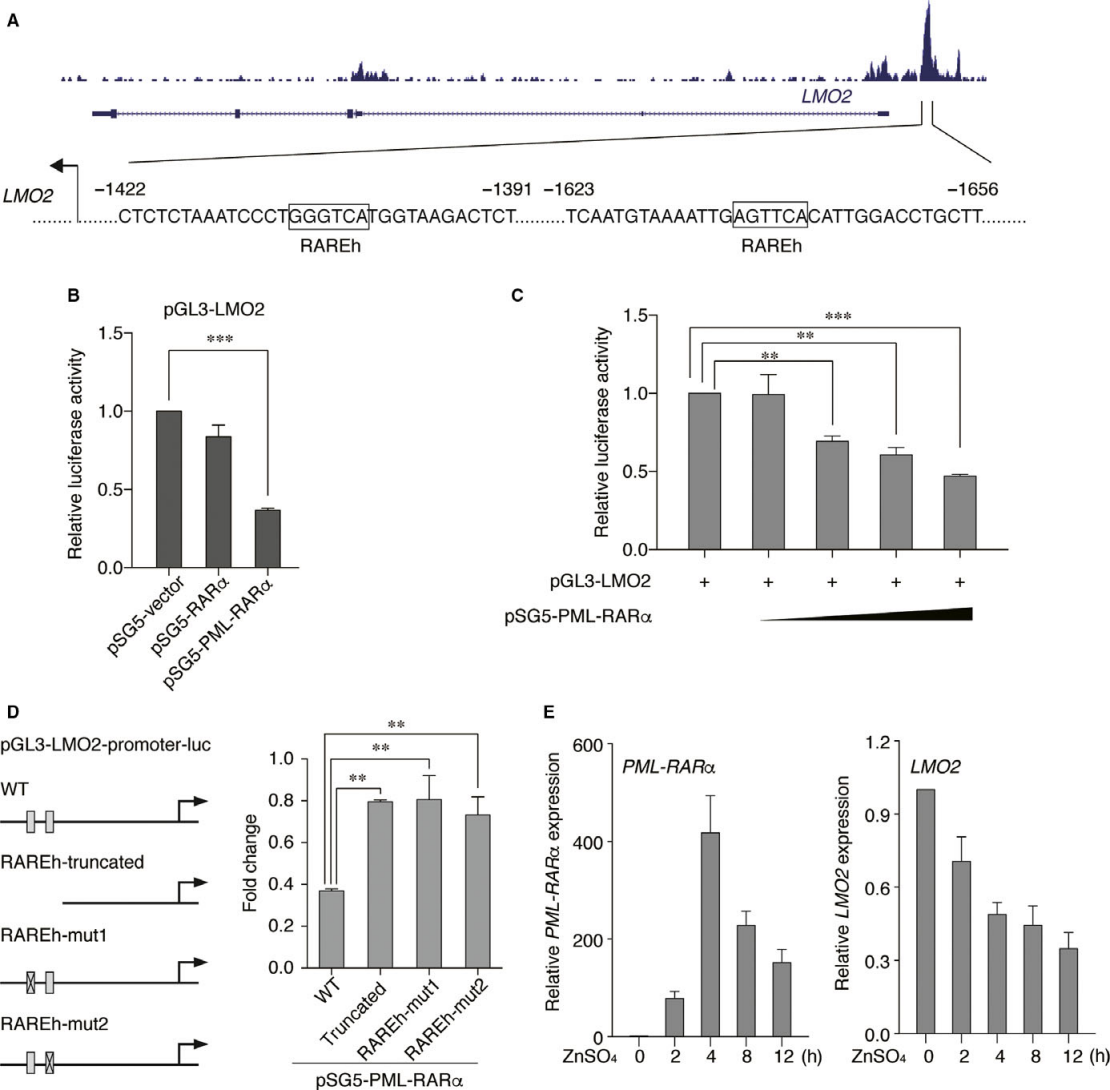
PML‐RARα down‐regulates the expression of *LMO2* through transcriptional repression of the *LMO2* distal transcript. (A) Schematic representation of the *LMO2* distal promoter. The half sites of retinoic acid responsive elements (RAREs) are defined using TRANSFAC with the core and matrix similarity. (B) PML‐RARα rather than wild‐type RARα repressed the transcriptional activity of the *LMO2* distal promoter. Luciferase reporter assays were performed in 293T cells. (‐) absence and (+) presence of the indicated plasmid. (C) The distal promoter activity of *LMO2* was repressed by PML‐RARα via a dose‐dependent manner. The *LMO2* distal promoter was transfected into 293T cells along with increasing amounts of the PML‐RARα expression construct. (D) Both RAREh sites were required for PML‐RARα‐mediated *LMO2* repression. Schematic representation of the *LMO2* distal promoter luciferase constructs including wild‐type, truncated construct and mutants (left panel). PML‐RARα failed to repress the luciferase activities of the truncated construct and mutants of the *LMO2* promoter. (E) *LMO2* expression was decreased after PML‐RARα induction in ZnSO_4_‐treated PR9 cells at a series of time‐points. RT‐PCR was performed to detect the expression of *PML‐RAR*α, the *LMO2* distal transcript and *GAPDH* respectively. Data represent the mean of three replicates ± SD, **P < 0.001; ***P < 0.0001

The next question we asked was whether such binding affected the transcription of *LMO2*. To answer this question, we first scanned the enriched motifs within the *LMO2* distal promoter. As shown in Figure 2A, we found two half sites of retinoic acid responsive elements (RAREs) with 300 bps of each other within the PML‐RARα binding peak. To determine if PML‐RARα represses *LMO2* transcriptional activity, we conducted promoter reporter assays using the distal promoter of *LMO2* in 293T cells, a non‐hematopoietic cell line. As illustrated in Figure 2B, after cotransfecting the PML‐RARα expression construct, we observed that the distal promoter activity of *LMO2* was transrepressed by PML‐RARα. Interestingly, wild‐type RARα had no impact on *LMO2* transcriptional activity, which was in line with the ChIP result that wild‐type RARα did not bind the *LMO2* distal promoter. Furthermore, PML‐RARα transrepressed *LMO2* distal promoter activity in a dose‐dependent manner (Figure 2C), demonstrating that *LMO2* was a transcriptional target of PML‐RARα. To further investigate if these two RARE half sites are involved in PML‐RARα‐mediated repression of *LMO2*, we generated three truncated or mutated *LMO2* distal promoters, one lacking these two RARE half sites and the other two with each mutated RAREh site, and then compared the luciferase activity upon PML‐RARα expression between the full length and truncated/mutated constructs. As shown in Figure 2D, PML‐RARα failed to repress the transcriptional activity of all three truncated/mutated constructs. The above observations suggested that PML‐RARα transrepressed the transcriptional activity of *LMO2* distal promoter through binding these two RARE half sites and both RAREh sites were required in this repression.

Next, to investigate if the expression of the *LMO2* distal transcript was subjected to the repressed transcriptional activity of the *LMO2* distal promoter, we performed qRT‐PCR in PR9 cells treated with ZnSO_4_ in a time series. As shown in Figure 2E, the expression level of the *LMO2* distal transcript was gradually decreased upon the PML‐RARα induction, indicating that the repression of the *LMO2* distal promoter by PML‐RARα resulted in the reduction in *LMO2* expression.

### 3.3 *LMO2* is expressed at a lower level in APL than in non‐APL AML subtypes

**Figure 3:**
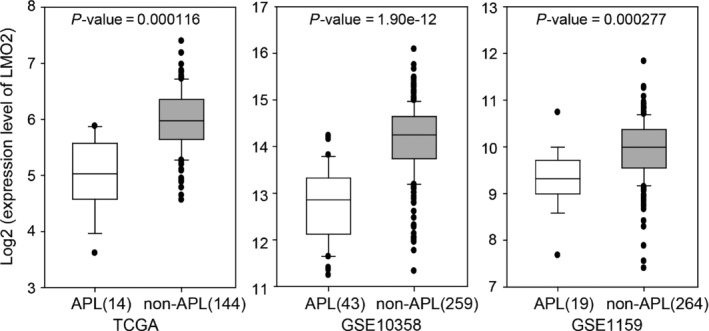
*LMO2* is expressed at a lower level in APL than in non‐APL AML subtypes. Three gene expression profiling data sets were retrieved, including TCGA,^20^ GSE10358 21 and GSE1159.^22^ The difference in *LMO2* expression between APL and non‐APL AML subtypes was assessed using the two‐tailed *t‐*test. The *P*‐values are shown in the panels

The above observations demonstrated that PML‐RARα repressed the *LMO2* expression via targeting the *LMO2* distal promoter, which indicated a negative correlation between PML‐RARα and LMO2 in APL. To further verify the correlation between *PML‐RAR*α and *LMO2* in a large population, we retrieved three data sets (TCGA, GSE10358 and GSE1159) on the expression profiling of 743 AML patients,^20^, ^21^, ^22^ including 76 APL patients and 667 patients with other AML subtypes. Using these data sets, we compared the *LMO2* expression values between APL patients and non‐APL AML patients. As shown in Figure 3, the large‐scale gene expression revealed that *LMO2* was expressed at a lower level in APL patients as compared with non‐APL AML patients, further confirming that *LMO2* expression was specifically down‐regulated with the expression of PML‐RARα in APL.

### 3.4 PML‐RARα interferes with erythroid differentiation through repressing *LMO2* in APL

**Figure 4:**
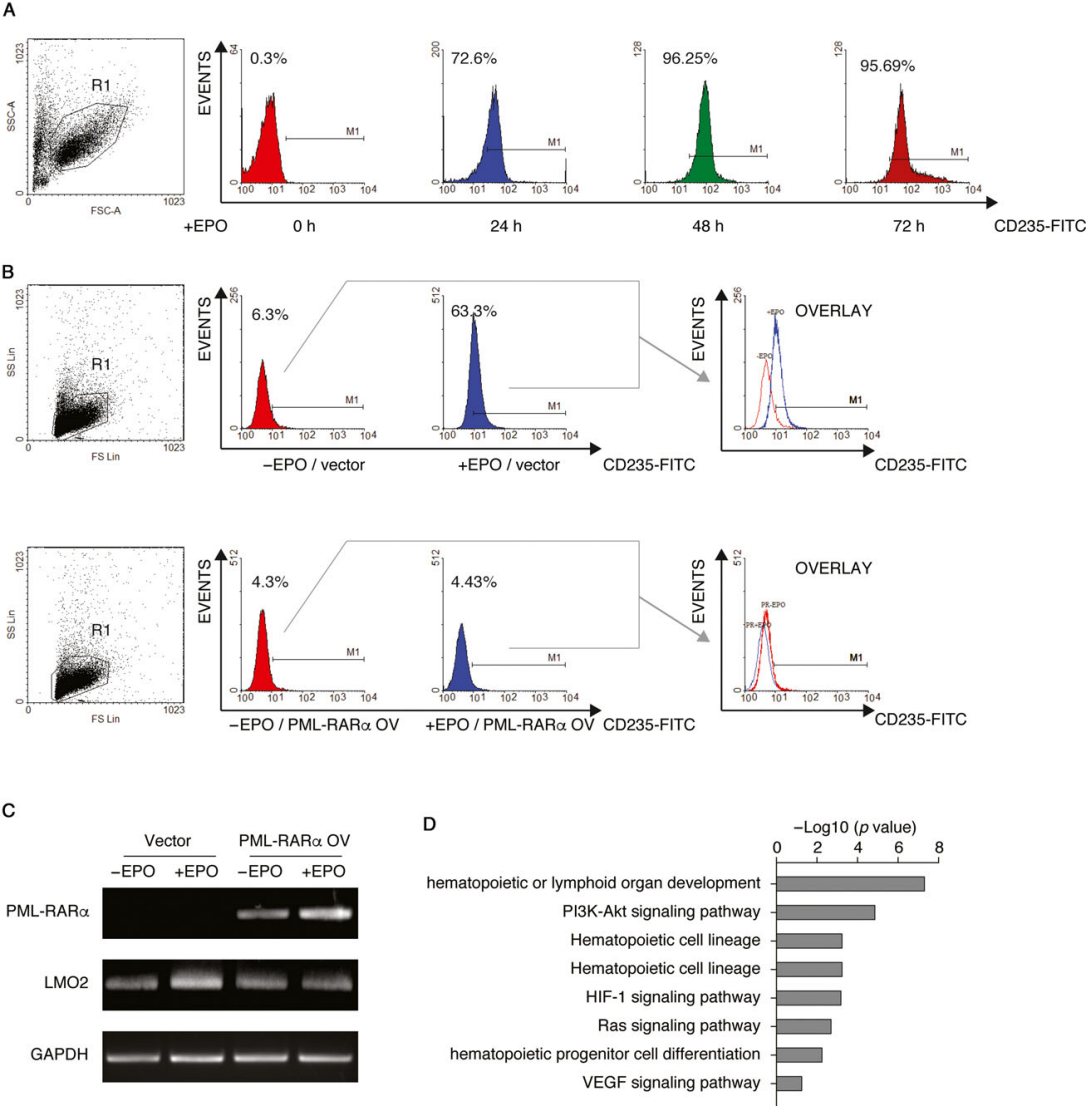
PML‐RARα interferes with erythroid differentiation through repressing *LMO2* by PML‐RARα. (A) Surface expression of the erythroid marker glycophorin A (CD235) was monitored by flow cytometry in UCB‐derived CD34 positive cells upon EPO treatment. (B) PML‐RARα interfered with EPO‐induced erythroid differentiation of CD34 positive cells. Ectopic expression of PML‐RARα decreased the induction of the cell surface expression of CD235 in EPO‐treated CD34 positive cells. (C) The up‐regulation of *LMO2* upon EPO treatment was repressed by PML‐RARα in CD34 positive cells. RT‐PCR was performed to check the expression of *LMO2* in CD34 positive cells and PML‐RARα‐overexpressed CD34 positive cells before or after treatment with EPO respectively. (D) Gene Ontology (GO) analysis of *LMO2* targets with differential expression between APL and non‐APL AML patients. The *P*‐values denote the significance of GO terms enrichment in the differentially expressed genes

Since *LMO2* plays a pivotal role in erythropoiesis, we postulated that the decreased *LMO2* expression caused by PML‐RARα might lead to the defective erythroid differentiation in APL. To test this hypothesis, we first treated UCB‐derived CD34 positive cells with EPO and then measured the expression of CD235 on the cell surface, which is a cell surface marker only expressing in mature erythroid cells. As shown in Figure 4A, the expression of CD235 was continuously up‐regulated after EPO treatment and the increase was maintained up to 72 hours, indicating that EPO was capable of stimulating the CD34 positive cells to differentiate into mature erythroid cells. Considering the efficiency of nucleofection, we selected the 24‐hour time‐point for the subsequent experiments. We further overexpressed the PML‐RARα expressing plasmid in CD34 positive cells to detect whether PML‐RARα would affect the EPO‐induced erythropoiesis. As illustrated in Figure 4B, we observed that EPO failed to induce the expression of CD235 on the surface of PML‐RARα expressing cells, suggesting that PML‐RARα might interfere with erythroid differentiation. Furthermore, we evaluated the mRNA levels of *PML‐RAR*α and the *LMO2* distal transcript to compare the *LMO2* expression before and after EPO treatment. We found that in control cells, EPO was able to up‐regulate *LMO2* expression. In contrast, in PML‐RARα expressing cells, *LMO2* expression had almost no change after EPO treatment (illustrated in Figure 4C), indicating that PML‐RARα inhibited EPO‐induced increase in *LMO2* expression. Taken together, our results suggest that PML‐RARα interferes with erythroid differentiation through inhibiting the expression of the *LMO2* distal transcript.

In the light of the observations that PML‐RARα interfered with erythropoiesis via *LMO2* suppression, we therefore assumed that PML‐RARα deregulated the *LMO2*‐dependent erythroid differentiation programme. To test this assumption, we retrieved ChIP‐Seq data using Lmo2 antibody in mouse haematopoietic progenitor HPC‐7 cell line^23^ and identified 4660 genes targeted by Lmo2. Among these target genes, 293 genes were differentially expressed between APL and non‐APL AML patients, which suggests that PML‐RARα deregulated the expression of these target genes through *LMO2* suppression in APL. GO and KEGG pathway analysis showed that the differentially expressed genes downstream of *LMO2* were enriched for pathways associated with haematopoietic development and haematopoietic progenitors differentiation as well as several erythropoiesis related signalling pathways (Figure 4D), such as Ras,^26^ PI3‐kinase^27^, ^28^ and hypoxic inducible factor 1 (HIF‐1) signalling pathways.^29^ Our results suggest that PML‐RARα disrupts erythroid differentiation programme through repression of *LMO2*, and thereby leads to the inhibition of erythropoiesis in APL.

## 4 DISCUSSION

Haematopoiesis is a tightly regulated process by which various lineage differentiation and commitment are controlled in a highly co‐ordinated manner. Leukaemia‐associated fusion proteins can disrupt this tightly controlled process through the aberrant transcriptional programmes, which results in a global differentiation block. The oncogenic PML‐RARα fusion protein dysregulates key regulators of normal haematopoiesis, such as PU.1,^30^ RUNX1^31^ and many others,^32^ as well as different pathways such as RAR signalling, thus resulting in the repression of critical myeloid gene expression and thereby contributing to the block at the promyelocytic stage. We show here that PML‐RARα also interfered with erythroid differentiation by directly targeting and repressing the expression of *LMO2* in the pathogenesis of APL.

Previous studies have described APL LICs from the different cell models. On the one hand, some studies suggest that APL LICs are myeloid committed progenitors. Interestingly, these studies are all based on the transgenic mouse models in which PML‐RARα expression is under the control of more committed myeloid specific promoters, such as CTSG, MRP8 and CD11b.^3^, ^4^, ^5^ It is therefore not surprising that the influence of PML‐RARα action in these models is restricted to the myeloid/granulocytic compartment. On the other hand, some studies performed on normal CD34+ Lin‐ cells suggest that PML‐RARα expression induces an APL phenotype possibly through three major sequential events, that is, differentiation commitment, rapid differentiation and promyeloid arrest.^6^ Furthermore, several studies have reported that the translocation of PML‐RARα occurs in pluripotent stem cells in APL patients.^7^, ^8^ These observations raise the possibility that PML‐RARα‐mediated cell transformation may be involved in different cell origins, although the true origin of leukaemia is still unknown because of the complexity of the disease origin and the limitations of current research methods.

Erythroid differentiation blocked by the expression of PML‐RARα has been demonstrated in several cell models.^6^, ^10^ For example, expression of PML‐RARα in CD34+/Lin‐ cells enables normal haematopoietic progenitor/stem cells to reach the promyelocytic level of differentiation but not to go further along the erythroid or the thrombocytic lineage, even if cells are cultivated in an adequate cytokine cocktail.^6^ Disrupted erythroid differentiation by the oncogenic fusion proteins is also associated with the pathogenesis of t(8;21) AML. AML1‐ETO is able to result in a gross inhibition of erythroid colony formation and thus inhibit early erythroid development.^33^ These observations strongly suggest a global differentiation block induced by the fusion proteins, which functions—at least for the erythroid lineage—already at a very early level, whereas the granulocytic precursors are blocked at a late stage of differentiation, such as at the promyelocytic level by PML‐RARα. We indeed provided the experimental evidence that PML‐RARα inhibited EPO‐induced erythropoiesis of human CD34 positive cells, which suggests a direct link between PML‐RARα and disruption of erythroid differentiation.

Erythropoiesis is orchestrated by a series of erythropoietic transcriptional factors. Many studies have demonstrated that these transcriptional factors promote erythroid development by forming the complex through the protein‐protein interaction.^12^, ^14^, ^15^ LMO2, similar to the well‐known erythropoietic transcriptional factor GATA‐1, is also regarded as the central factor in this transcriptional complex because it mediates the interaction between this complex and chromatin.^12^ *LMO2* is required to maintain at a relatively high expression level across erythroid development from haematopoietic stem cells to erythroblast.^34^ Down‐regulation of *LMO2* leads to inhibition of erythropoiesis. We found that PML‐RARα directly bound to the regulatory regions of *LMO2* and further repressed its expression, thus contributing to the disrupted erythroid differentiation in APL. Interestingly, in addition to LMO2, other factors in the transcriptional complex, including GATA‐1, LDB1, TCF3 and TAL1, showed no change or even higher expression in U937‐PR9 cells after PML‐RARα induction (Figure S2), suggesting the indispensable role of *LMO2* in erythropoiesis. Of note, we cannot exclude the possibilities that PML‐RARα can interfere with erythropoiesis at the protein‐protein interaction level. Indeed, PML‐RARα is able to interact with several haematopoietic specific transcription factors, such as AP‐1, GATA2, and PU.1.^24^, ^35^, ^36^ Mass spectrometry‐based screening can be applied to search for novel proteins that interact with PML‐RARα and are also involved in the regulation of erythropoiesis. The disruption of erythroid differentiation is also observed in other subtypes of leukaemia through impaired expression or activity of erythroid transcription factors by fusion proteins. For example, the AML1‐ETO fusion protein generated by t(8;21) has the capability to repress the expression of GATA‐1.^37^ Our findings emphasize the importance of *LMO2* in erythropoiesis and reveal a previously unidentified mechanism of defective erythropoiesis in APL, by which PML‐RARα specifically transrepressed *LMO2*, and thereby interfered with erythroid differentiation.

As part of our studies to determine how the expression of *LMO2* is disrupted by PML‐RARα in APL, we carried out an extensive analysis of the *LMO2* distal promoter region. Although three alternative promoters have been identified in the *LMO2* gene, our studies focused on the distal promoter since this region was specifically targeted and repressed by PML‐RARα. Indeed, it has been demonstrated that of the three promoters of *LMO2*, the distal promoter displays a hematopoietic restricted pattern, directing the hematopoietic‐specific expression of *LMO2*.^38^ Our luciferase assays and RT‐PCR results provide the experimental evidence that PML‐RARα transrepressed the expression of the *LMO2* distal transcript. Furthermore, we also demonstrated the requirement of two RAREh sites within the *LMO2* promoter in PML‐RARα‐mediated repression of *LMO2*. Our previous findings have shown that RAREh is significantly enriched in PML‐RARα binding sites and the RAREh sites are arranged in different orientations and with widely variable spacing in between.^24^ The two RAREh sites within the *LMO2* promoter were around 300 bp apart, raising the possibility that the two RAREh sites could be spatially close due to the high‐order structure of chromatin.

In addition to *LMO2* per se, we also looked at *LMO2* target genes and focused on the genes with differential expression between APL and non‐APL patients. Interestingly, we found that these genes were enriched in several signalling pathways critical for erythropoiesis. For instance, activation of PI3‐kinase is crucial for cell proliferation of erythroid progenitors.^28^ Moreover, PI3‐kinase/AKT signalling pathway is regarded as a mediator in EPO‐induced erythropoiesis through favoring *GATA‐1* transcription.^27^ Ras signalling pathway negatively regulates erythroid maturation by observing that overexpression of RAS blocks the differentiation of erythroid progenitor cells.^26^ HIF signalling is capable of promoting erythropoiesis at multiple levels, including regulation of EPO synthesis, enhancement of iron uptake and utilization as well as adjustment of bone marrow microenvironment for erythroid progenitor differentiation and maturation.^29^ Dysregulation of such erythropoiesis associated signalling pathways by repression of *LMO2* may have multifaceted effects on inhibition of erythropoiesis, further emphasizing the importance of repression of *LMO2* in erythroid deficiency in APL patients.

ATRA and ATO are two commonly and clinically used treatments applied for APL therapy. The therapeutic mechanisms of ATRA and ATO are different. ATRA mainly induces granulocytic terminal differentiation through transcriptional activation of the differentiation‐associated programme. ATO can rapidly degrade PML‐RARα fusion protein and induce the apoptosis of APL cells, thereby relieving the repression of genes targeted by PML‐RARα.^39^ In our study, we observed different changes in *LMO2* expression upon ATRA or ATO treatment in NB4 cells (Figure S1A and B). A similar observation has been found in our previous findings, in which *PSMB8*,* PSMB9* and *PSMB10* show response to ATRA but not ATO.^40^ *LMO2* expression showed no change or even further down‐regulation in ATRA‐treated NB4 cells (Figure S1B). The result observed upon ATRA treatment was reasonable, since miR‐223 is reported to repress *LMO2* expression, which is up‐regulated during the ATRA‐induced differentiation process from promyelocytes to neutrophils.^41^, ^42^ In contrast, ATO treatment could restore *LMO2* expression in NB4 cells (Figure S1A). Our data likely indicate that ATO but not ATRA has the ability to reactivate *LMO2* expression in APL cells.

Collectively, our findings identify *LMO2*, as a downstream target of PML‐RARα, whose dysregulated expression is associated with the failure of erythropoiesis in APL. Our data not only reveal a molecular mechanism of PML‐RARα‐mediated erythropoiesis inhibition but also provides evidence that PML‐RARα has broad impacts on multiple lineages of blood cells rather than myeloid lineage only.

## CONFLICT OF INTEREST

The authors declare no conflict of interest.

## AUTHOR CONTRIBUTION

XWY designed the study, performed experiments and wrote the manuscripts; YT, PW, HZ, MZ and XJZ performed experiments; KKW designed the study, interpreted the results and wrote the manuscript. All authors read and approved the final manuscript.
