# Supplementary figures and images for: PML‐RARα interferes with erythropoiesis by repressing LMO2 in acute promyelocytic leukaemia
Source: J Cell Mol Med. 2018 Oct 15;22(12):6275–84. doi: 10.1111/jcmm.13917 (PMC6237603; doi:10.1111/jcmm.13917)

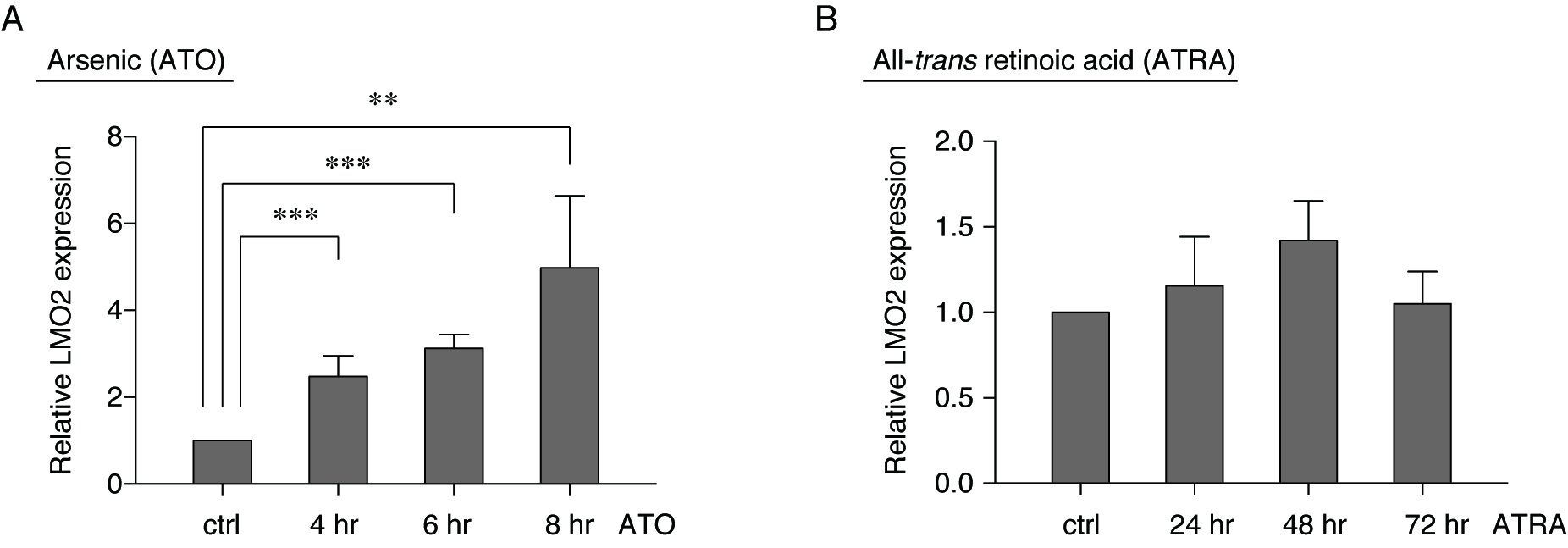

Supplement: Supplementary file 1 [file JCMM-22-6275-s001.tif]

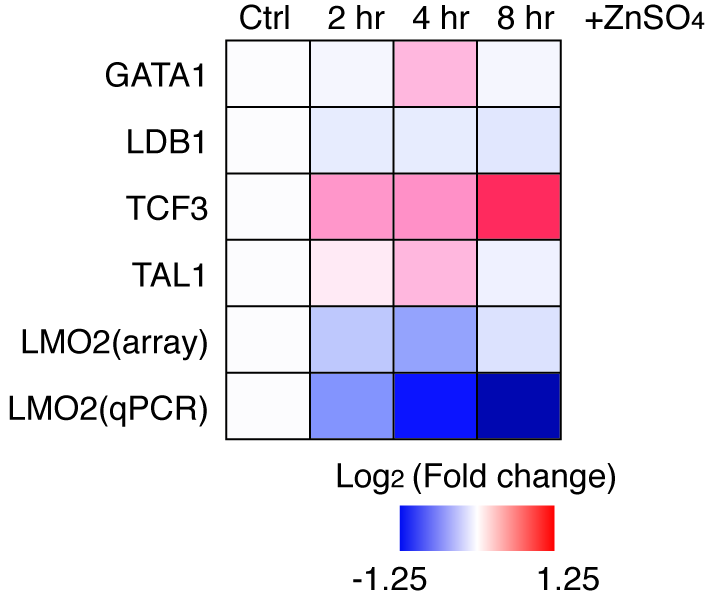

Supplement: Supplementary file 2 [file JCMM-22-6275-s002.tif]
